# Conditional guided generative diffusion for particle accelerator beam diagnostics

**DOI:** 10.1038/s41598-024-70302-z

**Published:** 2024-08-19

**Authors:** Alexander Scheinker

**Affiliations:** Applied Electrodynamics Group, Los Alamos National Laboratory, Los Alamos, NM 87545 USA

**Keywords:** Free-electron lasers, Statistics, Characterization and analytical techniques

## Abstract

Advanced accelerator-based light sources such as free electron lasers (FEL) accelerate highly relativistic electron beams to generate incredibly short (10s of femtoseconds) coherent flashes of light for dynamic imaging, whose brightness exceeds that of traditional synchrotron-based light sources by orders of magnitude. FEL operation requires precise control of the shape and energy of the extremely short electron bunches whose characteristics directly translate into the properties of the produced light. Control of short intense beams is difficult due to beam characteristics drifting with time and complex collective effects such as space charge and coherent synchrotron radiation. Detailed diagnostics of beam properties are therefore essential for precise beam control. Such measurements typically rely on a destructive approach based on a combination of a transverse deflecting resonant cavity followed by a dipole magnet in order to measure a beam’s 2D time vs energy longitudinal phase-space distribution. In this paper, we develop a non-invasive virtual diagnostic of an electron beam’s longitudinal phase space at megapixel resolution (1024 × 1024) based on a generative conditional diffusion model. We demonstrate the model’s generative ability on experimental data from the European X-ray FEL.

## Introduction

Particle accelerators provide intense high energy charged particle beams for a wide range of scientific studies at otherwise inaccessible length, time, and energy scales. Free electron lasers (FEL) generate bright flashes of coherent light at femtosecond time scales which is incredibly useful for structural biology^1^. FELs have been utilized for a wide range of biological studies including protein crystallography^2–4^, with a recent demonstration of single protein-based diffraction from a 14 nm diameter sample^5^. Two-color experiments with polarization control has enabled the use of FELs as tools for chiral recognition during photolysis^6^. FELS have been used to image viruses^7^, to study the structure and dynamics of macromolecules^8^, and FELs have been used to study matter in extreme conditions^9^. Utilizing FELs as femtosecond light sources has also enabled time-resolved site-specific investigations for understanding and benchmarking ultrafast photochemistry^10^. The data used in the current work was collected at the European X-ray FEL (EuXFEL)^11^. The EuXFEL is one of the most advanced FEL facilities in the world, capable of accelerating up to 5000 electron bunches per second up to energies of 17.5 GeV, with the FEL undulator producing hard X-rays at up to 14 keV with pulse energies of up to 2.0 mJ. The EuXFEL has been utilized for a wide range of scientific studies. Recent work at the EuXFEL includes the study of ribosome molecules^12^, the development of crystal-based photon energy calibration techniques for FELs^13^, the development of advanced single-particle X-ray diffractive imaging techniques^14^, laser-driven dynamic compression experiments for fast formation of nanodiamonds^15^, studies of ultrafast demagnetization induced by X-ray photons^16^, the development of novel single X-ray pulse-based 3D atomic structure reconstructions^17^, and for ultrahigh resolution X-ray Thomson scattering^18^.

In FELs, photocathode properties are crucial as they define the initial conditions of the electron beams which are then accelerated and used to produce the FEL light. The improvement and development of advanced FEL photocathodes is a lively area of research including a wide range of studies on photocathode technology^19–23^. Another area of intense FEL research is the development of non-destructive characterization methods for the FEL light pulses themselves. This is challenging as the pulses can be only 10s of femtoseconds in duration, but their characterization is crucial to fully understand the FEL-based imaging process and to verify the properties of the produced light. Towards these efforts, recently AI methods have been developed for online characterization of ultrashort X-ray free-electron laser pulses themselves^24^.

**Figure 1:**
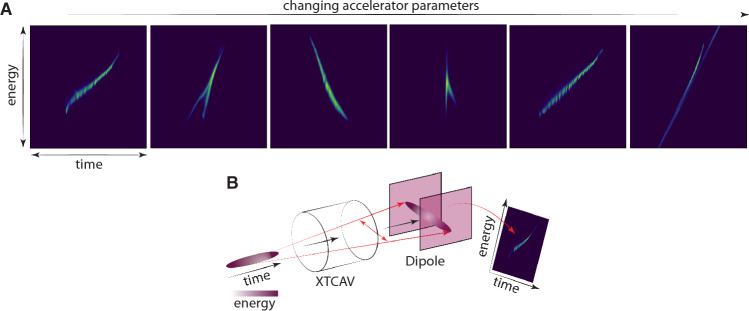
(**A**) Examples of longitudinal phase space measurements of electron beam at the EuXFEL for various accelerator components settings. (**B**) An overview of the destructive measurement process with the beam first sheared by a transverse deflecting RF cavity followed by energy-based dispersion of the beam with a dipole magnet.

After the electrons are produced at the photocathode and before they pass through the undulator to create intense pulses of light, the dynamics of intense electron bunches are influenced by complex collective effects such as wakefields, space charge, and coherent synchrotron radiation, making it difficult to control and tune beam properties using model-based approaches. Precisely shaping the 2D energy vs time longitudinal phase space distribution of the electron beam relies on an ability to measure that distribution in detail. The state of the art method for such measurements utilizes an x-band transverse deflecting cavity (XTCAV) to measure the beam. The XTCAV streaks the electron bunch, translating longitudinal position to transverse position. The sheared bunch is passed through a vertical dipole causing an energy-dependent curvature of the electron trajectory. The beam, which has now been significantly spread in terms of longitudinal position (*z*) and energy (*E*), then impacts a scintillating screen and the generated light intensity is recorded, providing a measurement of the (*z*, *E*) longitudinal phase space density which captures both longitudinal bunch current profile and energy distribution^25^. Several examples of such measurements taken at the European X-ray FEL (EuXFEL) are shown in Fig. 1. The images shown are 1024 × 1024 pixels with a time resolution of 1.5 fs/pixel (because the electrons are traveling at near light speed that translates into 4.5 μm/pixel), the energy resolution is 20 keV/pixel, the bunch charge is 0.25 nC, and the bunch energy is 150 MeV.

The main limitation of a XTCAV-based measurement is that it is an invasive procedure which destroys the beam that is being measured and therefore that same beam cannot be accelerated further for experimental applications. Furthermore, in many facilities (such as the EuXFEL) choosing whether to send the beam off to a diagnostic section or to allow the beam to continue accelerating is a lengthy tuning procedure. It is not possible to simply switch back and forth at the push of a button. Therefore the initial accelerator section essentially runs in two different modes. First, the XTCAV diagnostics section is used for initial tuning, then the beam is only accelerated downstream without further use of the XTCAV.

It would be valuable to measure the detailed time vs energy distribution of the electron beam near the beginning of the accelerator at all times non-invasively, both to provide a detailed understanding of the electron beam’s characteristics and also to use that information as input to online physics models which could then realistically estimate the beam’s dynamics through subsequent accelerator sections. Such diagnostics enable automatic real-time control of the beam’s longitudinal phase space distribution. For example, at the LCLS FEL the first demonstration of adaptive ML was used for automatic shaping of the longitudinal phase space of the electron beam by combining adaptive feedback with deep learning for time-varying systems^26^. These days, various machine learning (ML)-based methods for particle accelerators, including for use as virtual diagnostics have been studied for many accelerator applications. For example, neural networks are being used for uncertainty aware anomaly detection to predict errant beam pulses^27^.

In this paper, virtual diagnostic represent a large family of techniques which cover a broad spectrum of fully non-invasive methods to give virtual images of beam information which is not directly measured to techniques which rely on a combination of invasive measurements together with advanced algorithms to provide such measurements faster than otherwise possible. For example, one early example of a non-invasive virtual diagnostics was demonstrated at the FACET plasma wakefield accelerator, where an online physics model was adaptively tuned using non-invasive energy spread spectrum measurements in order to track the time-varying electron beam’s profile and longitudinal phase space, which would otherwise require a destructive TCAV-based measurement^28^. The use of a simple dense networks was then studied in^29^ for longitudinal phase space predictions, but the quality of the images was limited with clearly visible artifacts even for very low-resolution 100 × 100 pixel images.

Both the accuracy and resolution of ML-based virtual diagnostics were greatly increased by the use of convolutional neural networks which naturally learn spatial correlations by working directly on high-resolution images. For the EuXFEL, a convolutional neural network-based approach has been developed for generating high-resolution longitudinal (*z*, *E*) phase-space images of the electron beam^30^. Adaptive neural networks using advanced feedback control algorithms^31^ for adaptive latent space tuning of autoencoders have been developed to provide virtual 6D diagnostics of charged particle beams^32^, and these adaptive ML methods have been shown to increase the robustness of generative predictions far beyond the span of the training data, for tracking unknown time-varying beams^33^. Adaptive convolutional neural networks have also been designed for inverse problems that map downstream beam measurements back to the initial beam distribution^34^. Convolutional neural networks have also been combine with destructive beam measurements to develop extremely fast virtual diagnostics for 4D tomographic phase space reconstructions^35^. Neural network-based methods have also been developed for predicting the transverse emittance of space charge dominated beams^36^. Convolutional neural networks and clustering algorithms have also been developed for predicting the longitudinal phase space of FEL beams and also to cluster these images to highlight patterns within the data^37^. Recently, very interesting methods have also been studied for phase space reconstructions based on normalizing flows^38^.

**Figure 2:**
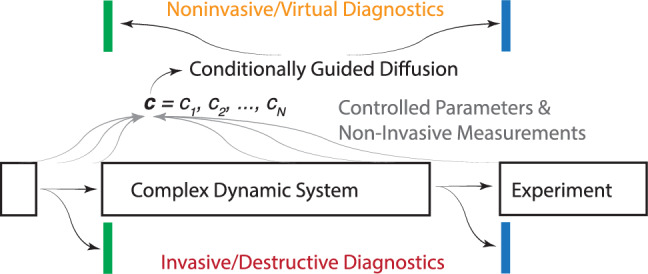
A general high-level overview of using conditional diffusion as a generative model that provides a non-destructive detailed view of the state of a complex system based on any available non-invasive diagnostics.

In this paper, the first diffusion based approach to non-invasive high resolution beam diagnostics is introduced. The diffusion-based model is developed for imaging the time vs energy longitudinal phase space distribution of a charged particle beam, and demonstrated on the European XFEL for accurately predicting the distributions of a diverse set of bunch profiles over a wide range of accelerator settings. Although this method is focused on a particle accelerator application, it is a very general approach, which can be used for any complex dynamic system for which it would be beneficial to replace invasive or destructive diagnostics of the system’s state with virtual diagnostics which must rely on low-dimensional non-invasive measurements or parameter set points. Such a general setup is shown in Fig. 2, where the goal is to develop virtual non-invasive diagnostics of the states of a complex dynamic system which complement invasive/destructive diagnostics. The main idea is to collect all non-invasively measured beam and system properties and use them to guide a conditional diffusion approach to generate a high resolution virtual view of those otherwise destructive measurements.

## Methods

**Figure 3:**
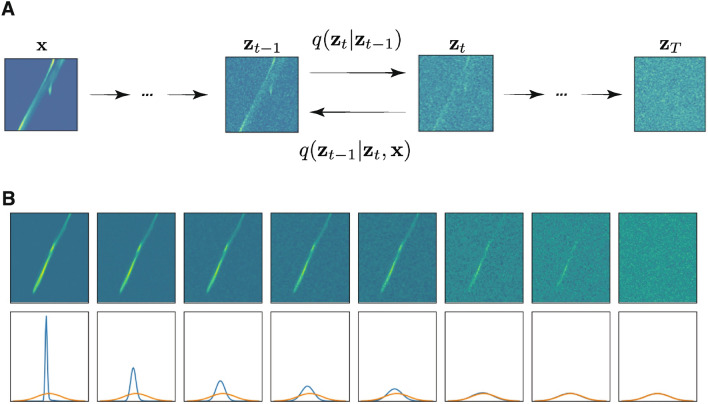
(**A**) The forward diffusion process of a sample $\textbf{x}$ is shown as it transforms to latent variables $\textbf{z}_t$ which gradually approach a Gaussian distribution. (**B**) A $T=1000$ step diffusion process is shown at each 125 steps along with a histogram of the image pixel values at each step (blue) next to a mean zero unit variance Gaussian (yellow).

Generative models based on diffusion utilize a gradual denoising approach inspired by statistical thermodynamics for modeling complex distributions^39^. This approach was then further developed for the generation of high resolution images^40–43^. Diffusion-based generative models have now become the state-of-the-art for generating high resolution images, especially when the images have a wide variety. The generative ability of diffusion-based models has made them powerful tools for a wide range of scientific applications^44^, such as conditional generation of hypothetical new familes of superconductotrs^45^, for brain imaging^46^, for various bioengineering applications^47^, for protein structure generation^48^.

The diffusion-based method used in this work is based on a modified version of the approach described in^40^. In our approach we add an additional conditional input vector along with the time embedding to perform guided diffusion which maps specific accelerator conditions to beam images. Conditional methods are a powerful approach to improve the predictive abilities of machine learning models by allowing a single model to learn over a wide range of data than what would otherwise be done by several individual models for specific tasks, as was recently demonstrated in conditional models outperforming other approaches for anomaly detection in particle accelerators^49^.

For completeness, we briefly outline the generative diffusion theory and refer readers to^40^ for details and proofs. The generative diffusion process works by adding noise to an image $\textbf{x}$ over a large number of steps *T* until the pixel values of the image closely resemble a mean zero unit variance Gaussian distribution as shown in the left to right flow of Fig. 3.

For an image $\textbf{x}$, the first step of the diffusion process is to create a noise-corrupted image $\textbf{z}_1$ as defined by

1 $$\begin{aligned} \textbf{z}_1 = \sqrt{1-\beta _1}\textbf{x} + \sqrt{\beta _1}\epsilon _1, \quad \epsilon _1 \sim \mathscr {N}(\epsilon _1|\textbf{0},\textbf{I}), \end{aligned}$$

with subsequent diffusion steps iteratively defined as

2 $$\begin{aligned} \textbf{z}_{t} = \sqrt{1-\beta _t}\textbf{z}_{t-1} + \sqrt{\beta _t}\epsilon _t, \quad \epsilon _t \sim \mathscr {N}(\epsilon _t|\textbf{0},\textbf{I}), \quad t\in \{2,\dots ,T\}. \end{aligned}$$

The noise schedule $\beta _t \in [0,1]$ with $\beta _1< \beta _2< \dots < \beta _T$ prescribes the variance for the additive unit variance Guassian noise $\epsilon _t$ at each step *t* which defines how quickly images are converted to pure noise. This diffusion sequence forms a Markov chain with conditional distributions of the form

3 $$\begin{aligned} q(\textbf{z}_t|\textbf{z}_{t-1})=\mathscr {N}(\textbf{z}_t | \sqrt{1-\beta _t}\textbf{z}_{t-1},\beta _t\textbf{I}), \end{aligned}$$

which is convenient for sampling random diffusion steps *t* without having to re-run the entire chain as $\textbf{z}_t$ can be rewritten as

4 $$\begin{aligned} \textbf{z}_t = \sqrt{\alpha _t}\textbf{x} + \sqrt{1-\alpha _t}\epsilon _t, \end{aligned}$$

where $\sqrt{1-\alpha _t}\epsilon _t \sim \mathscr {N}(\epsilon _t|\textbf{0},\sqrt{1-\alpha _t}\textbf{I})$ now represents the total noise added to the image in moving from step 1 to step *t*, with $\alpha _t$ given by

5 $$\begin{aligned} \alpha _t = \prod _{\tau =1}^{t}(1-\beta _\tau ). \end{aligned}$$

Eq. (4) implies that

6 $$\begin{aligned} q(\textbf{z}_t|\textbf{x}) = \mathscr {N}(\textbf{z}_t|\sqrt{\alpha _t}\textbf{x},(1-\alpha _t)\textbf{I}), \end{aligned}$$

and therefore, since $(1-\beta _t)<1$, as $T\rightarrow \infty$ the terms $\alpha _t$ and $1-\alpha _t$ approach 0 and 1, respectively, and

7 $$\begin{aligned} \lim _{T\rightarrow \infty }q(\textbf{z}_T|\textbf{x}) = \mathscr {N}(\textbf{z}_T|\textbf{0},\textbf{I}), \end{aligned}$$

which means that any image is converted to a signal indistinguishable from mean 0 unit variance Gaussian noise. In practice values such as $T=1000$ are a good choice, which is also the number of diffusion steps used in this work. The noise schedule is chosen with endpoints as in^40^ with $\beta _t$ increasing linearly from $10^{-4}$ to 0.02 over $T=1000$ steps. While nonlinear noise schedules were proposed in^42^, the authors pointed out that they were mostly beneficial for lower resolution images, and that was confirmed in this work as a cosine noise schedule performed similarly to the linear one.

In order to generate images, the model must learn to run backwards, undoing the diffusion process. For a given image $\textbf{x}$, Bayes’ rule and the Gaussian change of variables identity allow us to write the conditional probability $q(\textbf{z}_{t-1}|\textbf{z}_t,\textbf{x})$ which describes one reverse step of the diffusion process, as:

8 $$\begin{aligned} q(\textbf{z}_{t-1}|\textbf{z}_t,\textbf{x}) = \mathscr {N}\left( \textbf{z}_{t-1} \left| \frac{1-\alpha _{t-1}}{1-\alpha _t}\sqrt{1-\beta _t}\textbf{z}_t + \frac{\sqrt{\alpha _{t-1}}\beta _t}{1-\alpha _t}\textbf{x}, \frac{\beta _t(1-\alpha _{t-1})}{1-\alpha _t}\textbf{I} \right. \right) . \end{aligned}$$

By rewriting Eq. (4) as

9 $$\begin{aligned} \textbf{x} = \frac{1}{\sqrt{1-\beta _t}}\textbf{z}_t - \frac{\sqrt{\beta _t}}{\sqrt{1-\beta _t}}\mathbf {\epsilon }_t, \end{aligned}$$

and plugging that into the mean value of the prediction in Eq. (8), the neural network $D_\theta (\textbf{z}_t,t,\textbf{c})$ can be effectively used to predict how to remove noise between iterative steps to restore the original image according to

10 $$\begin{aligned} \textbf{z}_{t-1} = \frac{1}{\sqrt{1-\beta _t}}\left[ \textbf{z}_t - \frac{\beta _t}{\sqrt{1-\alpha _t}}D_\theta (\textbf{z}_t,t,\textbf{c}) \right] + \sqrt{\beta _t}\epsilon , \quad \epsilon \sim \mathscr {N}(\epsilon | 0,1), \end{aligned}$$

where $\theta$ represents the neural network’s weights. At the final generation step, the image is created according to

11 $$\begin{aligned} \textbf{x} = \frac{1}{\sqrt{1-\beta _1}}\left[ \textbf{z}_1 - \frac{\beta _1}{\sqrt{1-\alpha _1}}D_\theta (\textbf{z}_1,t,\textbf{c})\right] . \end{aligned}$$

The diffusion model is therefore trained to predict the noise at each diffusion step *t* by sampling a random $\epsilon \sim \mathcal {N}(\epsilon |\textbf{0},\textbf{I})$ and then updating the neural network’s weights according to a descent of the gradient

12 $$\begin{aligned} \nabla _\theta \left\| \epsilon - D_\theta (z_t,t,\textbf{c}) \right\| ^2 = \nabla _\theta \left\| \epsilon - D_\theta \left( \sqrt{\alpha _t}\textbf{x}+\sqrt{1-\alpha _t}\epsilon ,t,\textbf{c}\right) \right\| ^2 \end{aligned}$$

**Figure 4:**
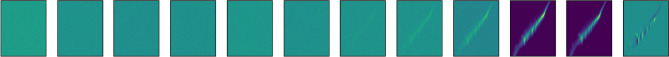
From left to right the first ten images show diffusion steps 100, 200, ..., 1000 followed by the true target image and finally the difference between the two.

One example of such diffusion-based image generation is shown in Fig. 4.

## Results

**Figure 5:**
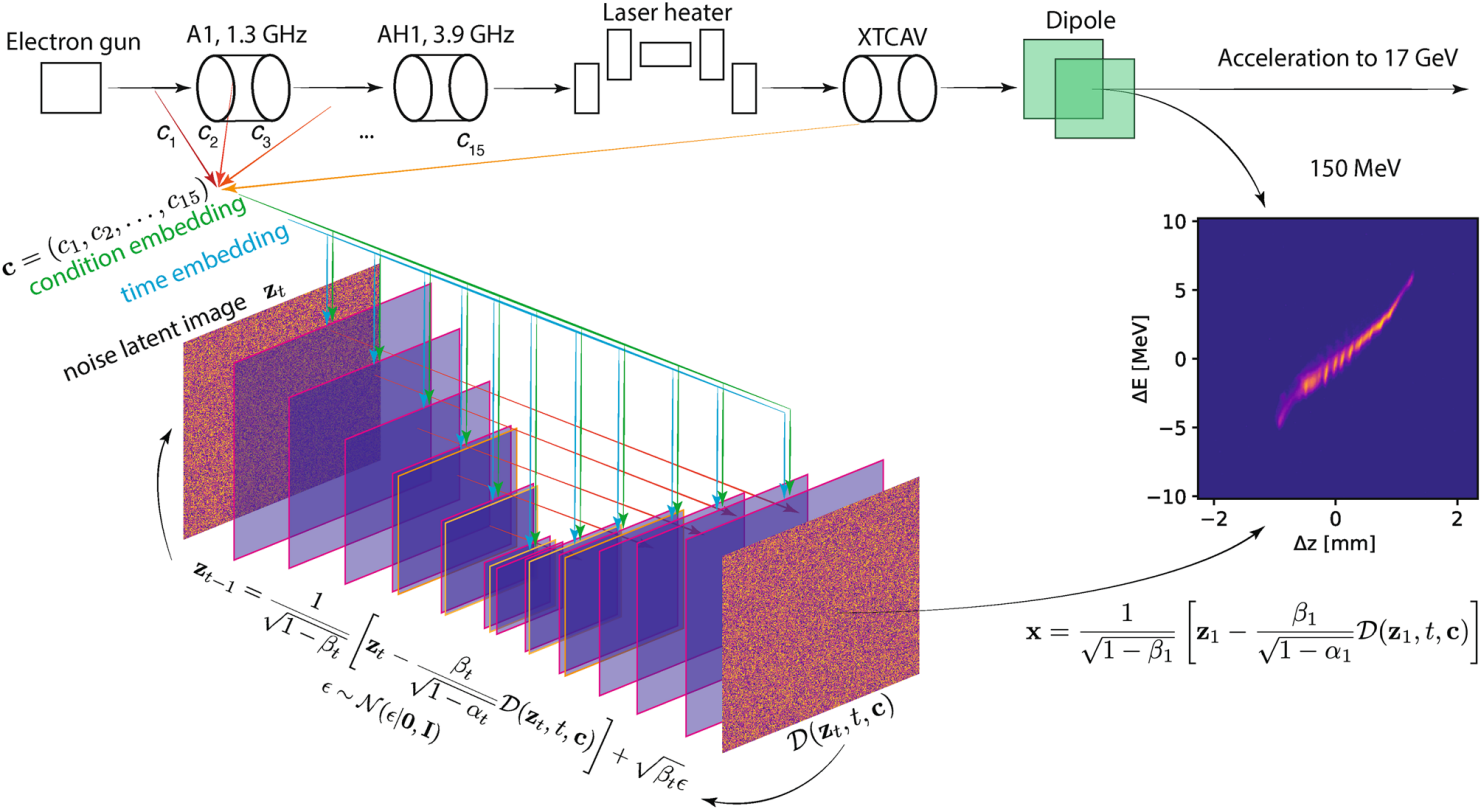
Conditional diffusion setup for generating XTCAV images of the beam’s (*z*, *E*) longitudinal phase space projection.

This work demonstrates that a conditionally guided generative diffusion process can be used to accurately generate unseen test data to give a non-invasive virtual high resolution view of the electron beam’s longitudinal (*z*, *E*) phase at the EuXFEL. In this approach, the conditional input vector is $\textbf{c}\in \mathbb {R}^{15}$ utilizing 5 accelerator parameter set points and 10 non-invasive beam-based measurements, as shown in Fig. 5. The overall architecture uses a U-net^50^, with the setup that was developed for the PixelCNN++^51^, which uses group normalization instead of regular normalization^52^. The initial input images are progressively downsampled by a factor of 2 at each step using convolutional layers with strides of 2, progressing from $1024\times 1024$ all the way down to $32 \times 32$ pixel images and swish activation functions are used throughout^53^. A sinusoidal position embedding, the same as that used for Transformers^54^, is used to specify which step *t* of $[1,2,\dots ,T]$ during the diffusion process is taking place. The conditional input $\textbf{c}$ is passed in together with the time-embedding as an additional channel at each step of the U-net. Self attention is applied at the two smallest resolution feature maps ($64\times 64$ and 32$\times$32).

The first 5 parameters of $\textbf{c}$ are settings of three energy chirps (energy vs time correlations designed for bunch compression) imposed by radio frequency (RF) resonant cavity fields onto the electron beam as it passes from the electron gun and is then compressed in length in the initial section of the accelerator. The curvature of this chirp is also controlled and so is the third derivative by using 3.9 GHz RF which is at the third harmonic of the EuXFEL’s overall 1.3 GHz RF system. The remaining 10 parameters are RMS *X* and *Y* beam centroid values measured at 5 locations between the injector and the XTCAV. The data used for this work was gathered at the EuXFEL by varying the first 5 parameters above within a wide range over a $\sim$36 hour period of time. For each of the 5 parameter set points the destructive XTCAV-based measurement of the beam was then performed and the BPM reading were also recorded. The BPMs were chosen as they are one of the most widely available non-invasive beam measurements in accelerators, and they would reflect any energy dependent trajectory changes in the beam if they were present from those parameter changes and they are non-invasive measurements that have the potential to capture some information about time variation of beam properties even when the 5 parameter set-points above are set at fixed values. From 11000 data points, 10000 were used for training and 1000 for testing. The EuXFEL is a large 5 kilometer long accelerator composed of thousands of components, whose properties do drift and change with time. Verifying this approach over unseen test data randomly sampled from the data collected over this time period verifies that the method is robust to time variation that takes place on several day time scales. A study of how robust this approach is to larger distribution shifts over longer periods of time will be the focus of future work. The expectation is that in order to make this method more robust to larger drifts over weeks/months instead of hours/days, will require the additional use of adaptive feedback algorithms as in^28,32–34^. One such adaptive diffusion approach is currently in development^55^. Compensating for distribution shift will also be aided by using more non-invasive beam-based measurements such as the BPMs described above.

**Figure 6:**
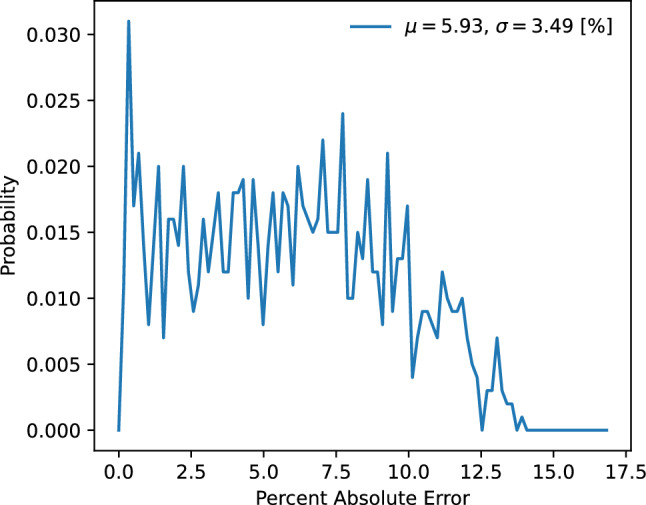
Error statistics for 1000 test images. The overall mean absolute percent error was 5.93 % with a standard deviation of 3.49 %.

**Figure 7:**
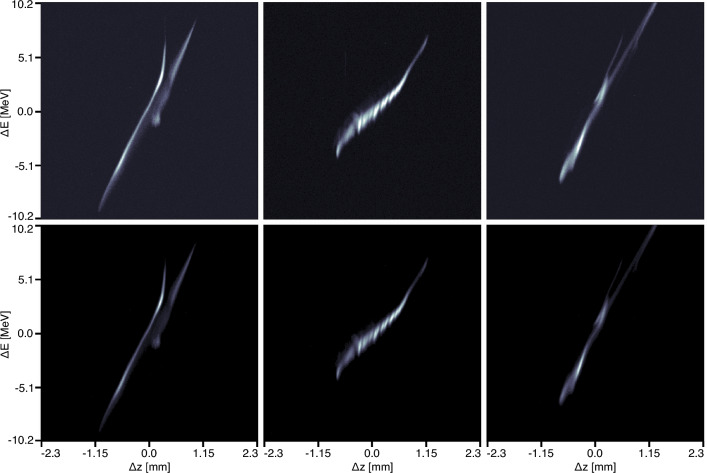
The top row shows a detailed view of 3 conditionally generated electron beam images at 1024$\times$1024 pixel resolution, based on a 1000-step diffusion process. The bottom row shows the target electron beam images.

To quantify the reconstruction accuracy the absolute percent error was calculated between generated images $\hat{Y}$ and their true measurements *Y* according to

13 $$\begin{aligned} E = 100\times \sum _i\sum _j \left| Y-\hat{Y} \right| / \sum _i\sum _j \left| Y \right| , \end{aligned}$$

where $i,j \in \{1,1024\}$ are the pixel locations within the images. Figure 6 quantitatively shows the generative error as defined in Eq. 13 for 1000 unseen test objects.

This generative conditionally guided diffusion approach results in an ability to create incredibly high resolution megapixel beam images for a very wide range of beams, which is exactly the application for which diffusion-based models are state-of-the-art. Figure 7 shows three detailed examples of very different very complex beams generated by the conditional diffusion process next to the true images.

**Figure 8:**
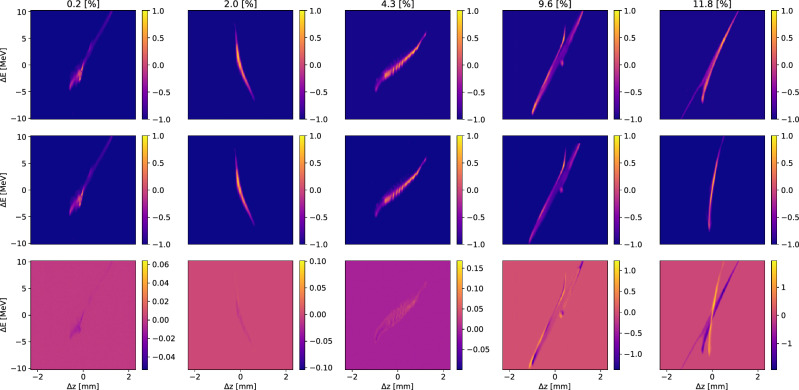
Detailed view of 5 reconstructed test images of increasing percent absolute error from left to right. The top row shows diffusion-generated images, the middle row shows the true measurements, and the bottom row shows their differences.

For a visualization of how these error levels correspond to image quality, Fig. 8 shows 5 examples from the test data set, of increasing error. It can be seen that up to $\sim 10$ % error the predictions are very accurate.

These results demonstrate that the conditionally guided diffusion model can serve as a highly accurate virtual diagnostic of the electron beam’s longitudinal phase space for a wide range of accelerator settings, without having to intercept and destroy the beam to measure it.

**Figure 9:**
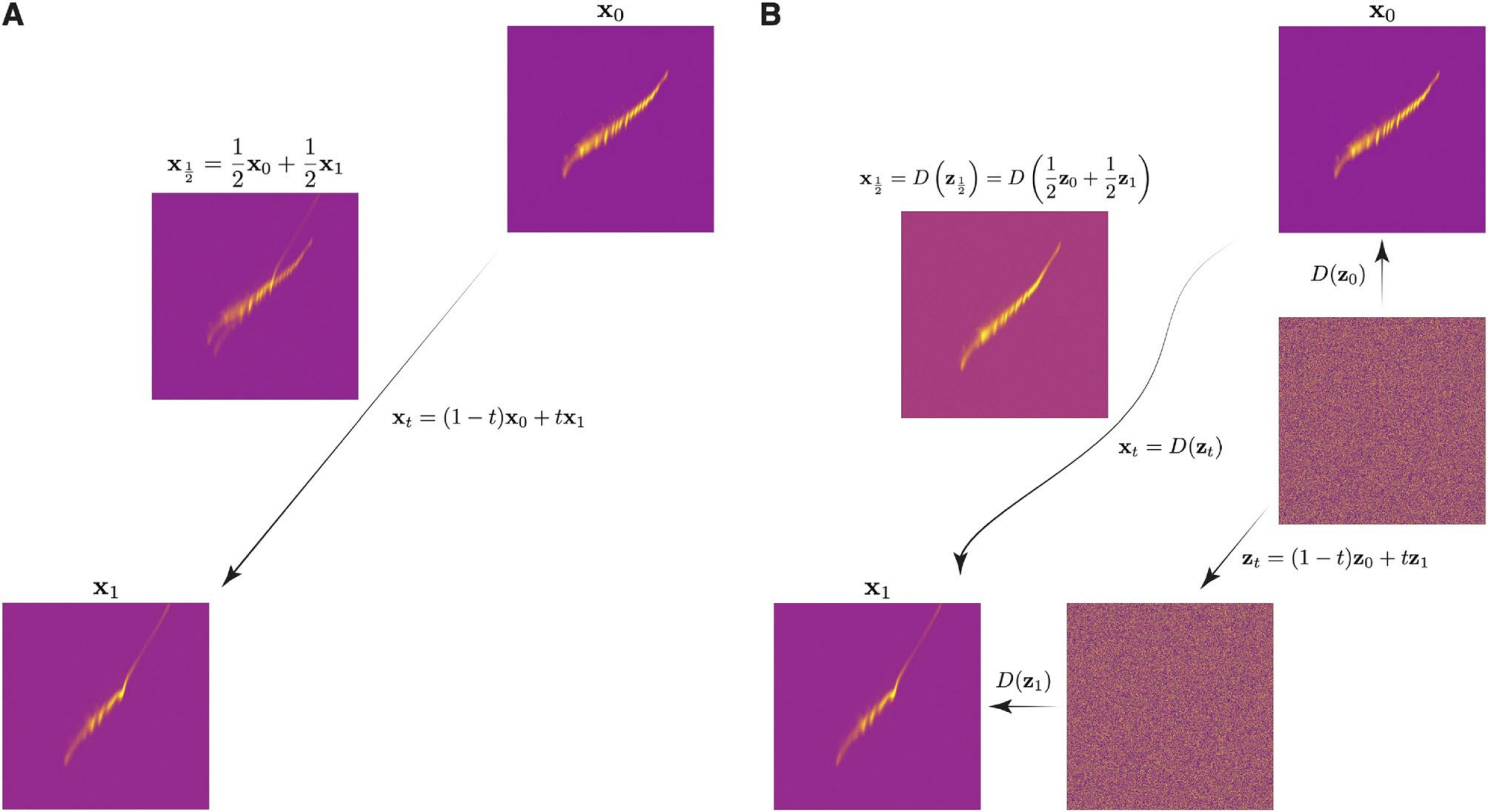
Left: Performing a simple linear interpolation between two images of the electron beam $(\textbf{x}_0,\textbf{x}_1)$ at two different accelerator settings results. Right: Performing linear interpolation in the latent space.

### Moving along the image manifold by latent space interpolation

Once the diffusion model has been trained to generate electron beam images associated with a wide range of accelerator settings, it is possible to smoothly move between various accelerator setups while generating realistic electron bunch distributions. Given two different accelerator setups $(\textbf{c}_0,\textbf{c}_1)$ and their associated electron beam images $(\textbf{x}_0,\textbf{x}_1)$, it would be useful to understand how the beam behaves at an intermediate state between these two. One simple naive way to attempt to approximate this is a linear interpolation between the two images of the form

14 $$\begin{aligned} \textbf{x}_t = (1-t) \textbf{x}_0 + t\textbf{x}_1, \quad t\in [0,1], \quad t: 0 \rightarrow 1 \quad \Longrightarrow \quad \textbf{x}_t: \textbf{x}_0 \rightarrow \textbf{x}_1. \end{aligned}$$

This method results in a non-physical weighted superposition of the two images, as shown on the left side of Fig. 9.

The trained diffusion model allows us to move between accelerator settings in a more physical way, as the network utilizes all of the training data in order to interpolate in a physically consistent way. In this approach, for the two images $(\textbf{x}_0,\textbf{x}_1)$ we first perform a conditional generation of the two images based on two random noise images ($\textbf{n}_0,\textbf{n}_1)$ and on their two conditioning vectors which correspond to accelerator settings $(\textbf{c}_0,\textbf{c}_1)$. Together we treat the noise-vector pairs as latent variables $(\textbf{z}_0,\textbf{z}_1)$. We now perform the same linear interpolation as above, but in the latent space directly according to

15 $$\begin{aligned} \textbf{z}_t = (1-t) \textbf{z}_0 + t\textbf{z}_1, \quad t\in [0,1], \quad t: 0 \rightarrow 1 \quad \Longrightarrow \quad \textbf{z}_t: \textbf{z}_0 \rightarrow \textbf{z}_1. \end{aligned}$$

At each time step along the latent path $\textbf{z}_t$ we can then perform conditional generation via the learned diffusion process in order to generate an image $\textbf{x}_t$ which is no longer a simple superposition of the two images, but rather is a true intermediate state that is moving along the learned image manifold

16 $$\begin{aligned} \textbf{x}_t = D(\textbf{z}_t) = D\left( (1-t) \textbf{z}_0 + t\textbf{z}_1\right) , \end{aligned}$$

as shown on the right side of Fig. 9.

It is important to note that when measuring the longitudinal phase space of a large particle accelerator’s electron beam using a transverse deflecting cavity and dipole, the image observed on a (transverse) screen inserted into the beam will be a convolution of the longitudinal and the transverse phase space. Therefore, there is potential that large changes in the transverse phase space will impact the TCAV-based measurements. This additional complexity adds another component to the overall distribution shift that is expected especially for longer periods of time. In order to measure and learn such relationships would require a larger parameter scan than what was performed here, in which transverse properties of the beam are intentionally varied over a wide range by, for example, scanning quadrupole magnet settings. As mentioned above, handling such time varying beam distribution shifts would also potentially be aided by incorporating adaptive feedback into this diffusion process.

### Uncertainty quantification

Because of the probabilistic nature of the diffusion mechanism, the trained conditional diffusion model acts like a conditional sampler from an underlying distribution. For a given conditional vector $\textbf{c}$ the model generates images $\textbf{x}_i$ according to

17 $$\begin{aligned} \textbf{x}_i \sim Pr(\textbf{x}_i,\textbf{z}_{1\dots T}|\theta _{1\dots T}, \textbf{c}), \end{aligned}$$

where $\theta _i$ are the weights of the trained U-net and their time embeddings. Therefore, for a given value of $\textbf{c}$ one may sample from the distribution repeatedly. This lends itself to a form of uncertainty quantification which encompasses both aleatoric and epistemic uncertainty as it reflects the inherent variability in the training data (aleatoric uncertainty) and the model’s ability to capture that variation, and it also represents some aspects of epistemic uncertainty which may be due to a lack of knowledge or data, because due to the various assumptions made in the diffusion process and the finite amount of training data, the model’s learned distribution is unlikely to perfectly match the true underlying data distribution.

This approach does not directly isolate aleatoric or epistemic uncertainty, but it does provide some kind of metric for quantifying the variability of the model’s outputs. Further studies on uncertainty quantification are planned for future work, such as utilizing more experimental data to compare the model’s output variation to the true known data variation. To demonstrate the approach here, two conditional vectors $\textbf{c}_1$ and $\textbf{c}_2$ are fixed and the model is then sampled 100 times for each, generating 100 version of the image $\textbf{x}_1$, $\{\textbf{x}_{i1}, i=1,\dots ,100\}$ associated with $\textbf{c}_1$ and of the image $\textbf{x}_2$, $\{\textbf{x}_{i2}, i=1,\dots ,100\}$ associated with $\textbf{c}_2$. The standard deviations are then calculated for each set of images

18 $$\begin{aligned} \sigma (\textbf{x}_j) = \sqrt{\frac{\sum _{i=1}^{100}\left( \textbf{x}_{ij}-\mu (\textbf{x}_j) \right) ^2}{100}}, \quad \mu (\textbf{x}_j) = \frac{1}{100}\sum _{i=1}^{100}\textbf{x}_{ij}. \end{aligned}$$

**Figure 10:**
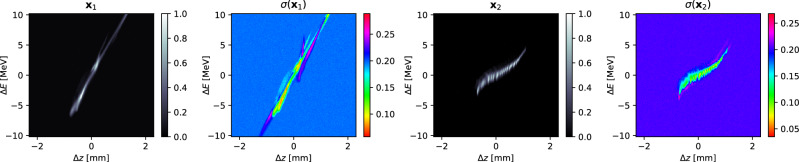
Two single examples of conditionally generated images are shown alongside the standard deviation of 100 versions of each sampled by the diffusion process.

Single examples of generated $\textbf{x}_1$ and $\textbf{x}_2$ alongside $\sigma (\textbf{x}_1)$ and $\sigma (\textbf{x}_2)$ are shown in Fig. 10.

## Discussion

This paper has demonstrated that conditionally guided generative diffusion models can be utilized as high resolution virtual diagnostics for charged particle beams. It was shown that the models can make accurate predictions for unseen test data within a very diverse set of measurements, and that the trained models can be used to smoothly traverse the learned latent embedding in order to interpolate between various accelerator settings in a physical way. Although this work was focused on high resolution 2D (*z*, *E*) longitudinal phase space predictions, because those are some of the most important measurements for FEL operations, this same approach can be used to model any of the beam’s projections, including all of the 15 unique 2D projections of a beam’s 6D phase space as was already done with autoencoder-based generative models^32,33^.

As discussed above, convolutional neural networks (CNN) are better suited and show greater performance (higher accuracy reconstructions) for image-based data than dense networks which recast images as long vectors and thereby destroy almost all of the local spatial relationships. Diffusion-based methods take advantage of this property of convolutional neural networks, and further utilize advanced U-net-based recursive architectures which share data at various image scales^50^, as well as attention layers to learn relationships in the lower dimensional latent representations of the U-nets^54^.

Beyond purely CNN-based approaches, the major benefit of using diffusion for virtual phase space diagnostics is that diffusion-based models have been proven to be the state-of-the-art for generating higher resolution images, especially when the images are highly varying as is the case for complex accelerator beams over a wide range of accelerator parameters as shown above, for class-conditional image synthesis^56,57^, and super-resolution methods^58^. Another major benefit of diffusion-based models is their ability to smoothly interpolate between very different images, by moving along the image manifold, which is again especially useful for exploring large configuration spaces of complex particle accelerators^59^.

Another bonus of diffusion-based models, as discussed above, is that like variational autoencoders they have a form of uncertainty quantification built in, which will be studied in more detail relative to accelerator data sets in future work. Beyond particle accelerators, such a conditionally guided diffusion process could be very useful for any complex dynamic system in which it would be beneficial to replace a destructive / invasive diagnostic of the system state with a virtual non-invasive virtual diagnostic.

## Data Availability

Datasets used and analyzed during the current study are available from the corresponding author upon reasonable request.
